# Manslaughter by Fake Artesunate in Asia—Will Africa Be Next?

**DOI:** 10.1371/journal.pmed.0030197

**Published:** 2006-06-13

**Authors:** Paul N Newton, Rose McGready, Facundo Fernandez, Michael D Green, Manuela Sunjio, Carinne Bruneton, Souly Phanouvong, Pascal Millet, Christopher J. M Whitty, Ambrose O Talisuna, Stephane Proux, Eva Maria Christophel, Grace Malenga, Pratap Singhasivanon, Kalifa Bojang, Harparkash Kaur, Kevin Palmer, Nicholas P. J Day, Brian M Greenwood, François Nosten, Nicholas J White

## Abstract

Fake artesunate could compromise the hope that artemisinin-based combination therapy offers for malaria control in Africa and Asia.

Falciparum malaria kills, and it particularly kills the rural poor. Artemisinin derivatives, such as artesunate, are a vital component of Plasmodium falciparum malaria treatment and control in the face of globally increasing antimalarial drug resistance. Since 1998 a worsening epidemic of sophisticated counterfeit “artesunate” tablets (containing no artesunate) has plagued mainland Southeast Asia (see Figure S1). In some countries, most of the available artesunate is fake [ 1–5].

Artemisinin derivatives are remarkably rapid in their antimalarial effects, and they are very well tolerated. So where these medicines are available, they are sought after. But as they are relatively expensive, a demand is created for cheaper versions amongst the poorest and most vulnerable people, upon whom the counterfeiters have preyed–with fatal results.

## Documented Death due to Fake Artesunate

The death of patients with untreated falciparum malaria, as a result of unwittingly taking fake artesunate, is hidden in the inadequately documented mortality statistics of the relatively voiceless rural poor. But there is no doubt that such deaths occur, and they are probably common.

In February 2005, a 23-year-old man presented with fever to a rural hospital in eastern Burma where he was diagnosed as having uncomplicated hyperparasitaemic falciparum malaria by microscopy (4.2% infected red blood cells). He was treated with oral artesunate, labelled as made by Guilin Pharmaceutical (Guangxi, People's Republic of China), 4 mg/kg once a day, the treatment of choice in this area. Since artemisinin derivatives have been used in this area, not one of 600 patients prospectively studied with ≥4% parasitaemia has died [ 6]. However, on the third night the young Burmese man became unconscious and was transferred to another hospital where he was found to be in a coma (Glasgow Coma Score = 3/15), with renal failure and a higher parasitaemia (5.5% infected red blood cells). He was perfused with intravenous fluids and received an injection of intravenous artesunate (2.4 mg/kg) and transferred to a third hospital where he died within 12 hours of arrival from cerebral malaria.

The original hospital sent the artesunate used to treat the patient for analysis by the Fast Red dye test [ 7]. We found that the artesunate was counterfeit. Based on the appearance of the counterfeit hologram affixed to the blister pack, it was identified as a Type 9 counterfeit artesunate (see Figure S1). Accurate mass measurements by direct time-of-flight mass spectrometry [ 8] revealed that the main active ingredient in this “drug” was paracetamol (acetaminophen), but that artesunate was also present in the tablet [ 9]. Quantification using high-performance liquid chromatography determined that the artesunate content was 10 mg per tablet, instead of the ∼50 mg of artesunate present in the genuine product [ 9].

The hospital where he was originally treated had, in good faith, recently purchased a large quantity of artesunate tablets. They were dismayed to learn from this death that their entire stock was fake. The village committee, which now had a clear idea of what was responsible for the man's death, was so angered by this avoidable death that they collected all the artesunate, fake and genuine, that they could find in local shops and destroyed it on a public bonfire in front of the whole village, to stress the lethal dangers of fake antimalarials.

## The Epidemic of Counterfeit Artesunate in Southeast Asia

Counterfeit artesunate continues to circulate on a vast scale in mainland Southeast Asia, where between 38% and 52% of “artesunate” blister packs sampled contain no active ingredient [ 1–5]. So far, only the Guilin Pharmaceutical brand of artesunate has been counterfeited. We make no apology for the use of the term manslaughter to describe this criminal lethal trade. Indeed, some might call it murder. Somewhere, people are directing a highly technical and sophisticated criminal trade. They are making tablets out of starch, chalk, and a variety of wrong active ingredients, such as erythromycin [ 9, 10], for a life-threatening disease that particularly affects the poor and underprivileged. The criminals are making these fakes in the full knowledge that their ineffective product might kill people who would otherwise survive their malaria infection.

There are now at least 12 different types of fake artesunate, classified by the sophisticated counterfeit holograms that are affixed to the blister packs (see Figure S1). Evidence suggests that production is on an industrial scale and from multiple sources; 100,000 counterfeit artesunate tablets were purchased from one large pharmacy [ 2].

This epidemic of a counterfeit, vital, life-saving medicine has received little practical attention over the last eight years, in comparison with the considerable efforts in other aspects of malaria control [ 3, 11]. In addition to unnecessary loss of life for profit, it has led to a loss of confidence in these very effective medicines and given rise to false reports of artemisinin resistance [ 1, 12]. Much more needs to be done in Asia to combat this scourge.

## The Risk to Western Travellers

This criminal activity is unlikely to remain a local difficulty, and there are serious implications from this major public health problem for the wider world beyond Asia that deserve attention. In the industrialised North, with carefully regulated trade, the implications are limited. However, especially as the artemisinin derivatives have a natural plant origin, tourists commonly buy them in the tropics as a standby treatment [ 13]. Indeed, Web sites encourage this practice [ 14], which is likely to be compounded by the availability of artemisinin derivatives on the Internet [ 15]. It is inevitable that counterfeit artesunate will seep into this trade.

We suggest that travel clinics should warn those going to the tropics of the potential dangers of buying such drugs. Unfortunately, in our discriminatory world, the unnecessary death of a tourist, journalist, or of a diplomat or military personnel, from a wealthy, influential country after self-medicating with fake artesunate, may be required to trigger the political will required to eradicate this lethal trade.

## Will Africa Be Next?

Of far greater concern is that counterfeits may follow in the wake of the genuine artesunate that is increasingly being imported for use in sub-Saharan Africa, where the burden of malaria is greatest. Since 2001, the World Health Organisation has recommended that malaria-endemic African countries should consider changing to artemisinin derivative– based combination therapy (ACT) as first-line malaria treatment. In the past two years, most countries in Africa (34 in 2004) have made this change [ 16]. Implementing this new policy will not be easy because of the high cost of ACTs and a temporary shortage of the plant raw material. It is estimated that 130 million courses of ACT will be used in Africa in 2006 [ 16]. High cost and shortage of ACT provide a favourable situation for the spread of fake artemisinins that could put the lives of thousands of African children at risk. There is already a thriving fake antimalarial drug industry in Africa [ 17], suggesting that it is highly likely that counterfeit artesunate or ACTs will follow in the wake of the genuine products, and in bulk.

**Figure 1:**
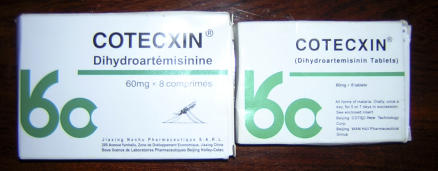
Genuine (Left) and Counterfeit (Right) Cotexcin (Dihydroartemisinin) from Tanzania (Photograph by Manuela Sunjio)

**Figure 2:**
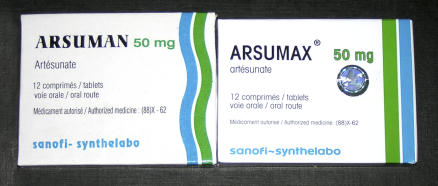
Genuine (Right) and Counterfeit (Left) Arsumax (Artesunate) from Cameroon (Photograph by Manuela Sunjio) Note that the genuine Arsumax bears a hologram from Guilin Pharmaceutical, which manufactures the tablets (see Figure S1).

Counterfeit dihydroartemisinin (60 mg per tablet; Cotexcin) was reported from Tanzania in 2001 [ 18], labelled as made by Beijing COTEC New Technology Corp, and containing no dihydroartemisinin or other active drug when analysed by thin layer chromatography and high-performance liquid chromatography ( Figure 1, collection and analysis of the sample by MS, CB, and PM). In 2005, counterfeit artesunate tablets, mimicking Arsumax (50 mg per tablet; Sanofi Synthelabo, Bridgewater, New Jersey, United States) were found in Cameroon ( Figure 2, collection and analysis of the sample by MS, CB, and PM). These were labelled as Arsuman manufactured by Sanofi Synthelabo, who confirmed that the packaging was counterfeit. On high-performance liquid chromatography analysis, these tablets did contain 50 mg of artesunate (MS, CB, PM), and this counterfeit is a look-alike copy of the genuine product. That at least two different counterfeit artemisinin derivatives have already been distributed in Africa is of considerable concern. Because of inadequate systems for the monitoring of the quality of antimalarial medicines, and because few have looked for it, counterfeit artesunate may already be widespread.

It is likely that, initially, most ACTs in Africa will be provided through the public health system where it should be relatively easy to control quality by purchasing only from established companies or from an international purchasing facility and by providing strong support to national quality control laboratories. However, the possibility of corruption within the national purchasing process cannot be excluded, as the potential financial gains for those involved could be very high.

In many parts of Africa, most patients with uncomplicated malaria obtain treatment from the private sector. Provision of free, or heavily subsidised, highly effective ACTs through public health facilities may reduce this proportion. However, it is likely that for the foreseeable future a substantial proportion of antimalarials used in Africa will be obtained through the private sector and it is here that the danger from fake artemisinins is greatest. Official promotional campaigns are likely to create immense demand for ACTs through the private system even though many potential users will struggle to meet their cost. In such a situation, introduction of a relatively inexpensive fake ACT product could lead to widespread usage of the fake drug with disastrous consequences.

## Preventing an Epidemic of Fake Artesunate in Africa

How might the spread of fake artesunate be prevented? Control of medicine importation is a first barrier of defence, but this is difficult to maintain and antimalarials are readily shipped across porous frontiers. There are at least 11 different brands of oral artesunate available in sub-Saharan Africa, including the genuine Guilin Pharmaceutical product. One of the authors was recently offered seven different artemisinin derivative brands in one small-town West African pharmacy—it is unlikely that all of these had been imported through official channels.

A second more radical option is to ensure that ACTs provided through the private sector are relatively inexpensive and locally affordable so that there is no financial advantage to looking elsewhere and thereby unwittingly purchasing a fake. This would require some form of central subsidy, as recently suggested in an Institute of Medicine (Washington D. C., United States) report [ 19]. This recommendation was made primarily to increase access to ACTs and to discourage monotherapy with artemisinins or the partner medicine (the co-drug in ACT) and thus to protect the ACT from the emergence of resistant parasites. Such an approach would have the additional advantage of discouraging the use of fake artemisinin-based medicines, as the counterfeit manufacturers would have little margin to make a profit. We strongly suggest that African countries and health organisations support such strategies to try to prevent counterfeit artemisinins from infecting Africa, to establish effective systems to carefully monitor their antimalarial drug supply, and to prepare to counter a problem that, as is evident from Asia, is very difficult to eradicate once established.

Of global concern for the future of malaria control, some counterfeit artesunate samples (Types 4, 10, and 11) recently collected in eastern Burma do contain small subtherapeutic quantities of artesunate (3.5–12.1 mg/tablet [ 9]). P. falciparum parasites with stable in vivo resistance to the artemisinin derivatives have not yet been described from the wild, but parasites with reduced in vitro sensitivity to artemether have recently been reported [ 20]. The in vivo exposure of parasites to low concentrations of artesunate from fake products will greatly increase the risk of the selection and spread of artemisinin resistant parasites, leading to the catastrophic loss of these essential medicines and an entirely avoidable failure of malaria control. We cannot afford to lose these drugs, as we have lost chloroquine and sulphadoxine–pyrimethamine—most current combinations depend on ACTs. In addition, the presence of small quantities of artesunate in tablets may mean that the Fast Red dye test [ 7], widely used for screening the quality of artesunate tablets, may give false positive results depending on how much artesunate is present in these fakes.

## Conclusion

Fake artesunate could compromise the hope that ACT therapy offers for malaria control in Africa and Asia. Fakes containing subtherapeutic amounts of artesunate could also result in the emergence and spread of resistance to the artemisinin drugs, shortening the useful life of these vital medicines. But this tragedy is avoidable, if there is sufficient political will. As global efforts to control malaria rely heavily on these drugs, these issues deserve overdue, urgent action to prevent a public health disaster in the malarious world.

## Supporting Information

Figure S1 Fake Artesunate Warning Sheet Number 4, April 2006 At least 12 different types of fake artesunate are being sold in mainland Southeast Asia. This warning sheet gives some key features to aid identification of these fakes.
(1.1 MB PDF).

## Abbreviations

ACT: artemisinin derivative–based combination therapy
