# Supplementary material for: Manslaughter by Fake Artesunate in Asia—Will Africa Be Next?
Source: PLoS Med. 2006 Jun 13;3(6):e197. doi: 10.1371/journal.pmed.0030197 (PMC1475657; doi:10.1371/journal.pmed.0030197)
Supplement: Supplementary file 1 [file pmed.0030197.sg001.pdf]

WARNING !!!! April 2006

AT LEAST TWELVE DIFFERENT TYPES OF FAKE ARTESUNATE ARE BEING SOLD IN MAINLAND SOUTH EAST ASIA

HERE ARE SOME KEY FEATURES TO AID IDENTIFICATION OF FAKE 'GUILIN PHARMA' ARTESUNATE BLISTER PACK HOLOGRAMS

BLISTER PACKS WITH FAKE HOLOGRAMS CONTAIN NO OR INSUFFICIENT ARTESUNATE AND ARE THEREFORE POTENTIALLY LETHAL

Genuine 'Guilin Pharma' Hologram

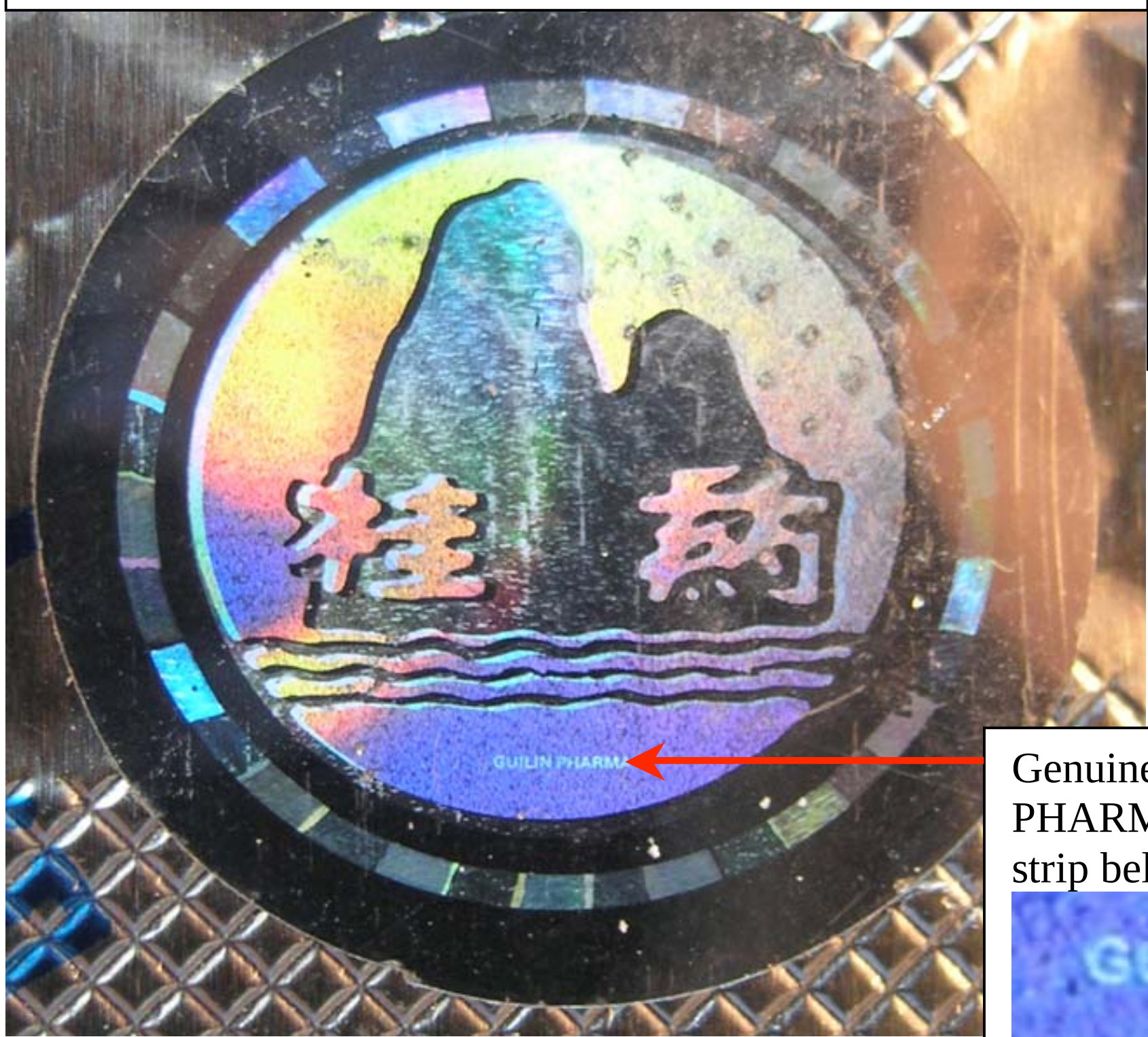

The photograph, left, is of the genuine hologram attached to the blister packs and packets of the genuine 'Guilin Pharma' artesunate. Below are pictures of 11 types of fake artesunate stickers & holograms with their distinguishing features. As hologram colour depends on the light, please do not use the colours printed here to judge whether artesunate is genuine or fake

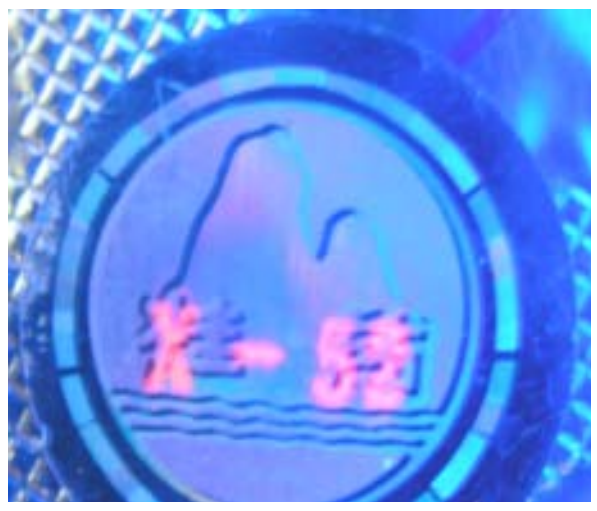

Genuine hologram under UV light with 'X-52' at mountain base

Genuine hologram has the legend 'GUILIN PHARMA' here. Visible with the naked eye, as a thin strip below the waves. Letters are ~ 0.1mm high

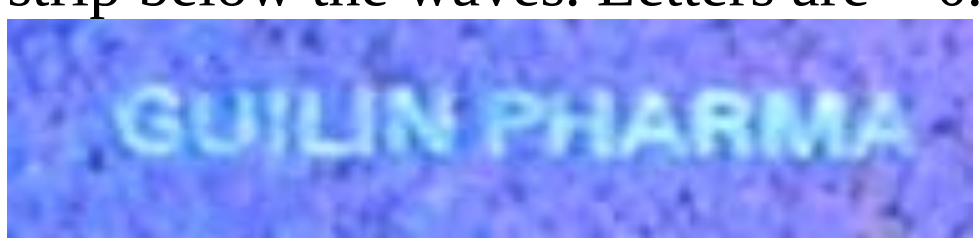

Guilin Pharmaceutical Co., Ltd. developed their hologram as a security device in response to the first type (**'Type 1'**) of counterfeit artesunate circulated which had no hologram attached. As a result, any 'Guilin Pharma' artesunate now circulating without a blister pack hologram is almost certainly counterfeit or expired. Subsequently, at least 11 different types of counterfeit 'Guilin Pharma artesunate' have been found in Southeast Asia [Burma (Myanmar), Cambodia, China, Lao PDR, Thailand (only on the western border) and Vietnam] with counterfeit stickers or holograms, and there are probably more types circulating. Genuine and counterfeit holograms are ~ 13 mm in diameter. The genuine and 'Type 10' holograms have 'X-52' visible across the mountains when viewed under UV light. Other features of the counterfeit artesunate that differ from the genuine product include – poor printing of bar codes on packets, poor printing of codes on blister packs, codes on blister packs printed with the font made of numerous small dots, poor stamping of code 'AS' and score line on tablets, raised edges of tablets, very hard tablets, blisterpack abbreviations in capital letters ('MFG' and 'EXP') and unusually late expiry date in relation to date of manufacture (>3years). Counterfeits tend to be cheaper than the genuine product.
This update has been issued following the discovery that counterfeit artesunate 'Types 4' and '9' contain small quantities of artesunate (~ 10mg) which will not be efficacious but may give misleading positive result on the Fast Red TR dye test and may facilitate the selection of artesunate-resistant malaria parasites. Although, regrettably, the counterfeiters have targeted the 'Guilin Pharma' product, it is possible that other fake artesunate products exist in Asia. Therefore, drug regulatory authorities should be vigilant and regularly monitor the quality of artesunate available on the market. If you are uncertain as the authenticity of any artesunate sample based on the characteristics of the packaging, please check by chemical analysis of the tablet

Counterfeit Artesunate – Type 2

A foil sticker. Not a true hologram; dull, not diffractive when tilted in the light. Not well crafted. 'Artesunate Tablet' printed on blisterpack

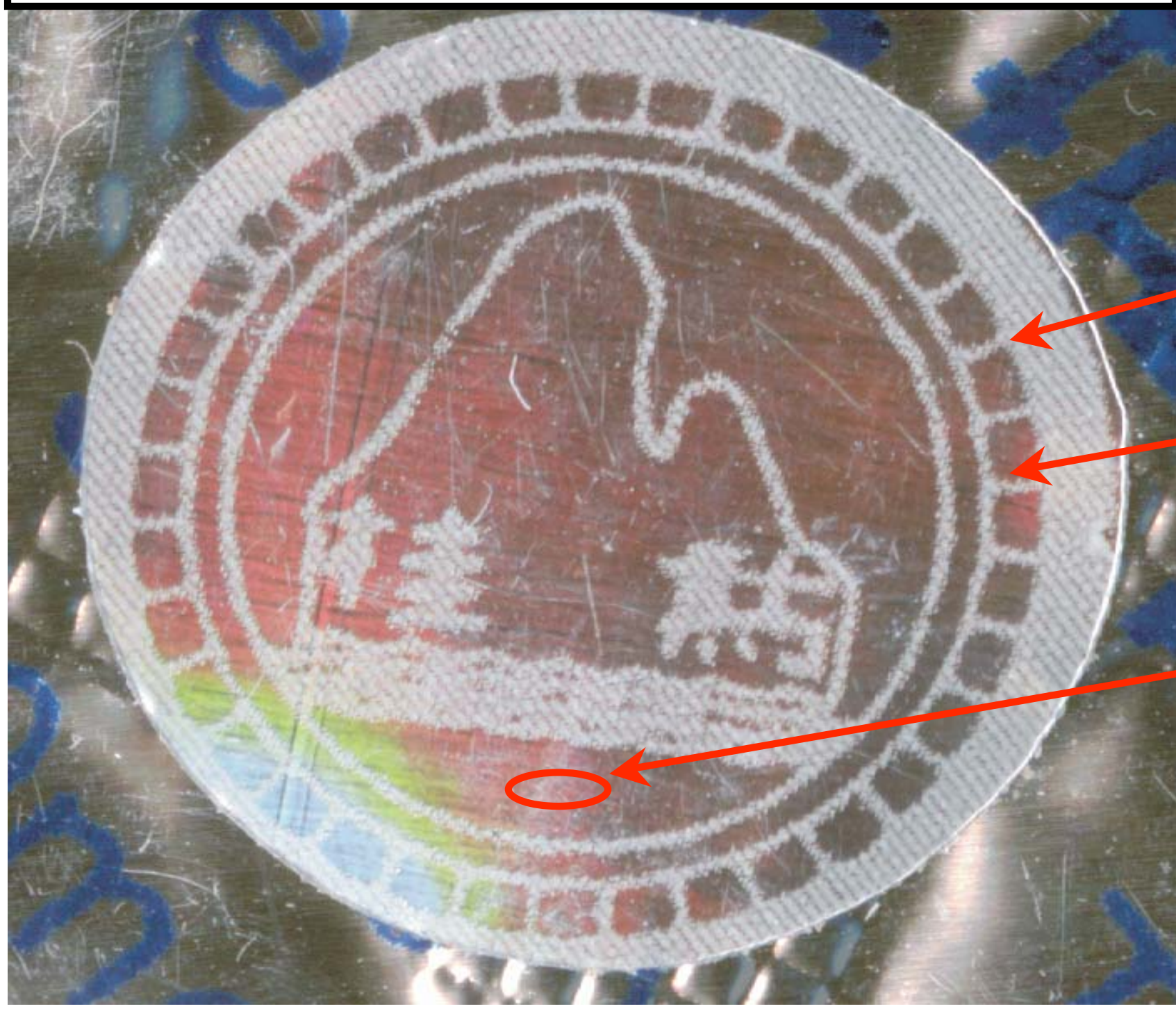

Grey outer ring

Inner ring rectangles do not vary in colour

No 'Guilin Pharma' legend

Blister packs seen with this fake sticker are printed with batch numbers 970901, 980201 or 980502.

Counterfeit Artesunate – Type 3

A hologram, but lacks the Guilin Pharma legend below the waves and the mountain outline differs. The left slope of the mountain meets the ring. The printing on the blister pack is not clear. 'Artesunate Tablet' printed on blisterpack

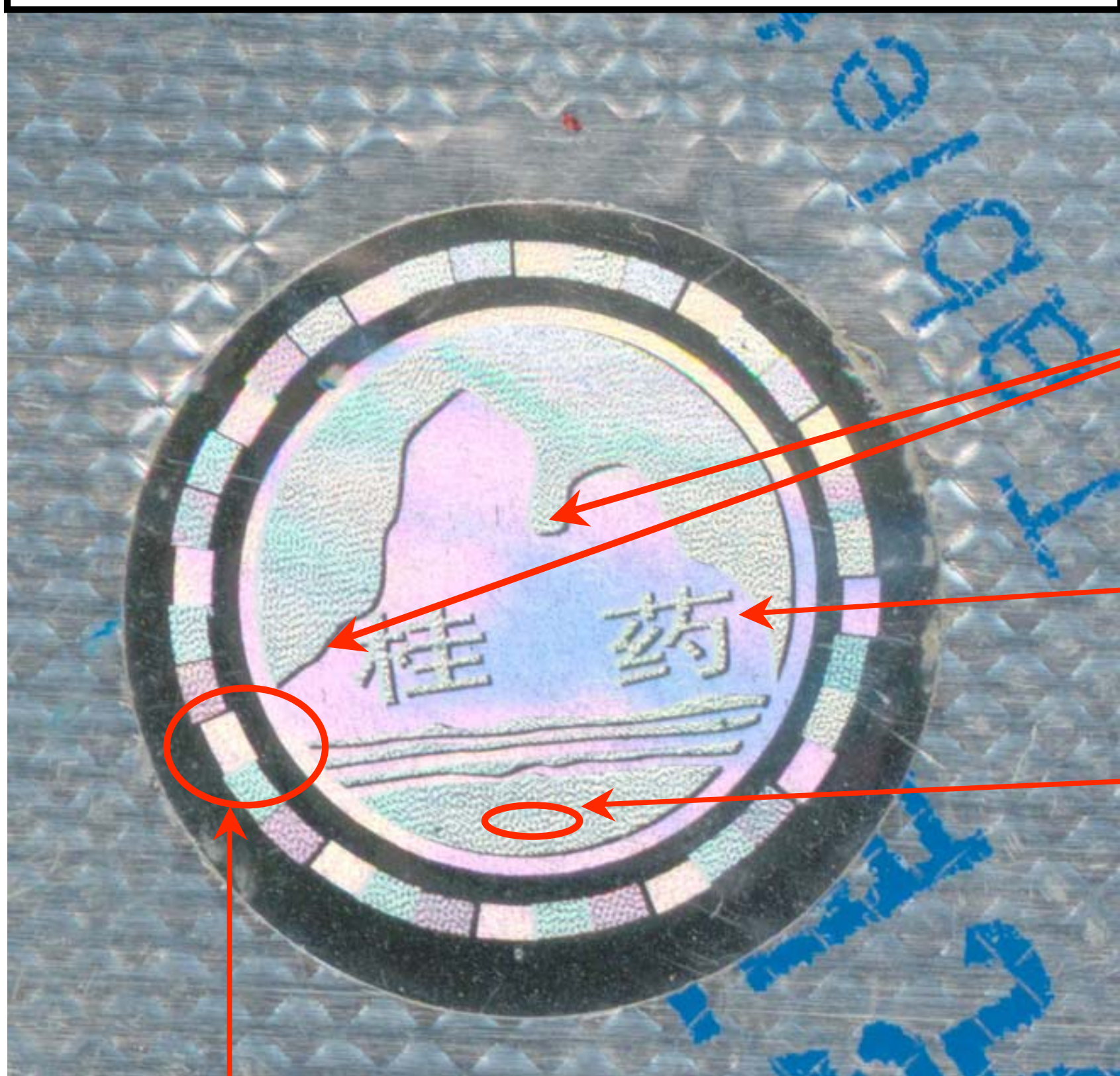

Mountain outline differs

Pictogram made of straight bars

No 'Guilin Pharma' legend

Blister packs seen with this fake hologram are printed with batch number 000902

Ring 'blocks' out of alignment

Counterfeit Artesunate – Type 4

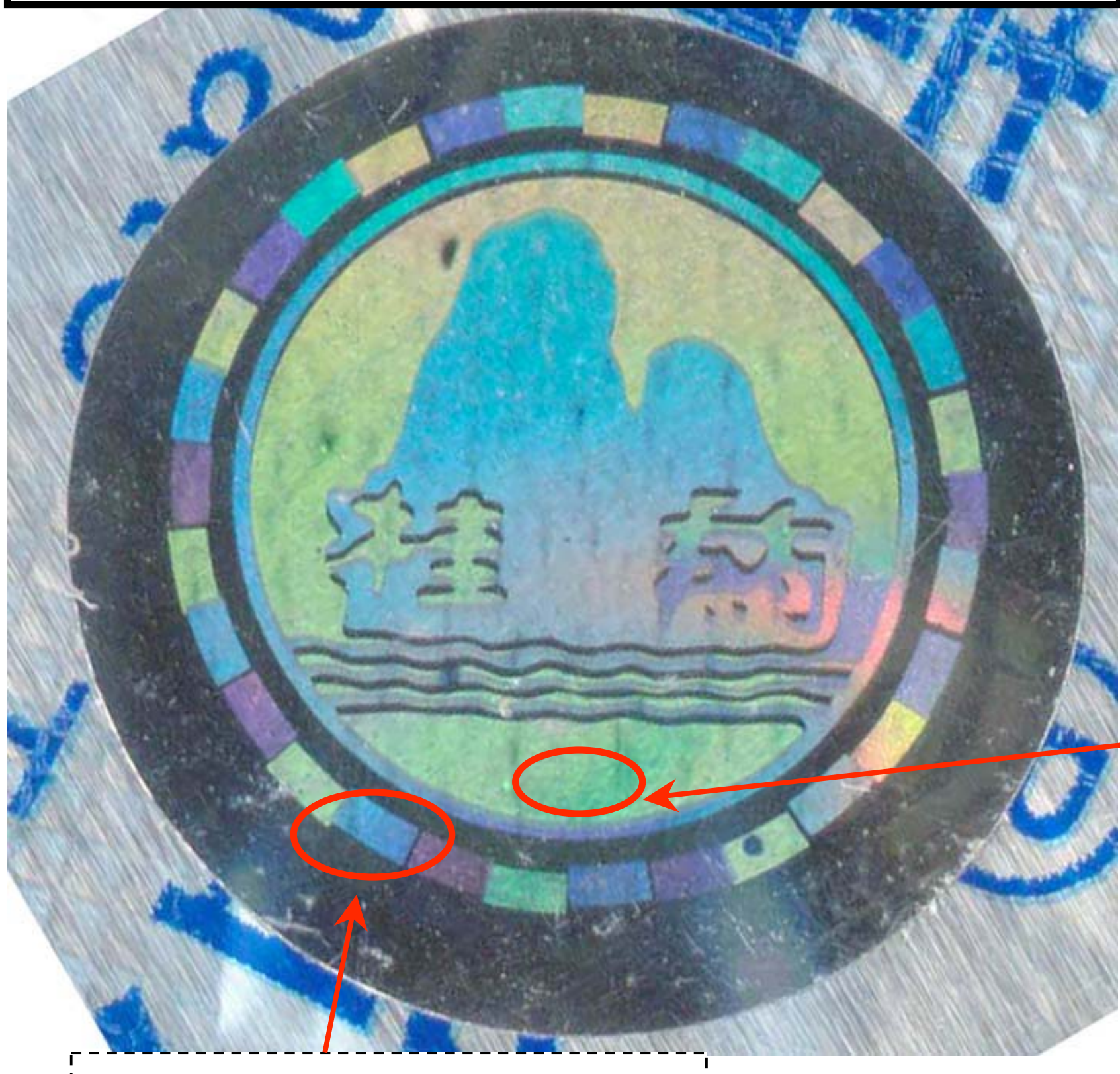

Ring 'blocks' out of alignment

No 'Guilin Pharma' legend

A hologram – mountain outline similar to that on the genuine hologram, but lacks the 'Guilin Pharma' legend below the waves. The printing on the blisterpack is clear. 'Artesunate Tablet', 'Artesunate Tablets' or Artesunate Tablte' (see below) printed on blisterpack.

Traces of artesunate found in tablets

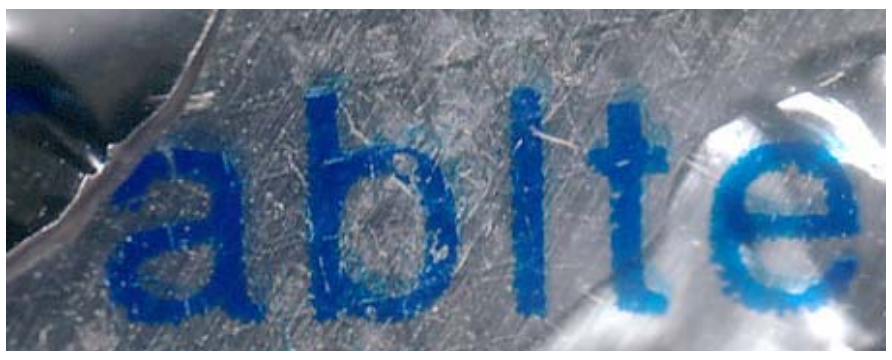

Blister packs seen with this fake hologram are printed with batch numbers 010901, 021001 or 020305.

Counterfeit Artesunate – Type 5

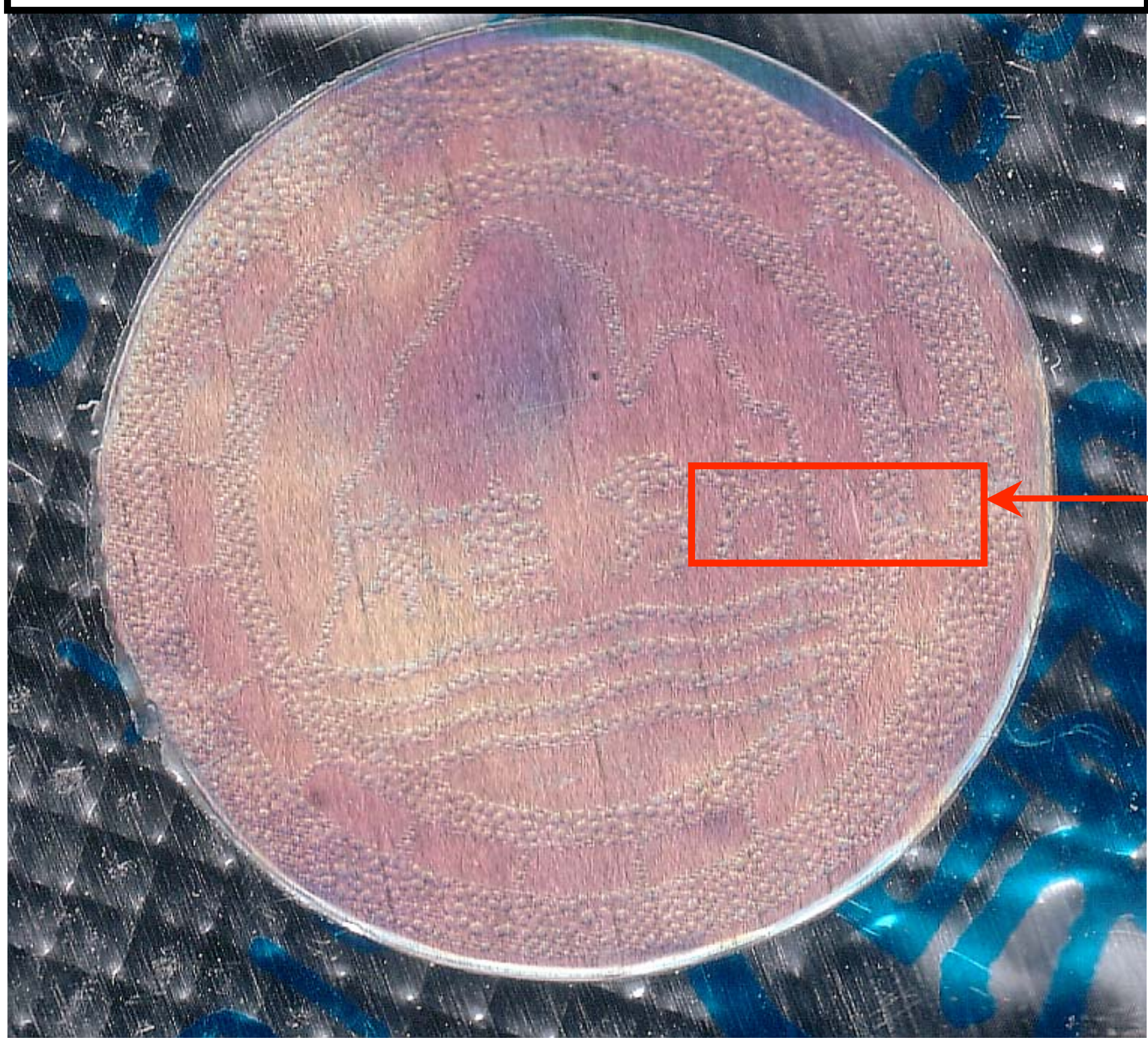

Foil sticker, not a hologram, with pattern composed of thousands of dots copying the genuine Guilin Pharma artesunate hologram pattern. Easily distinguished. 'Artesunate Tablet' printed on blisterpack

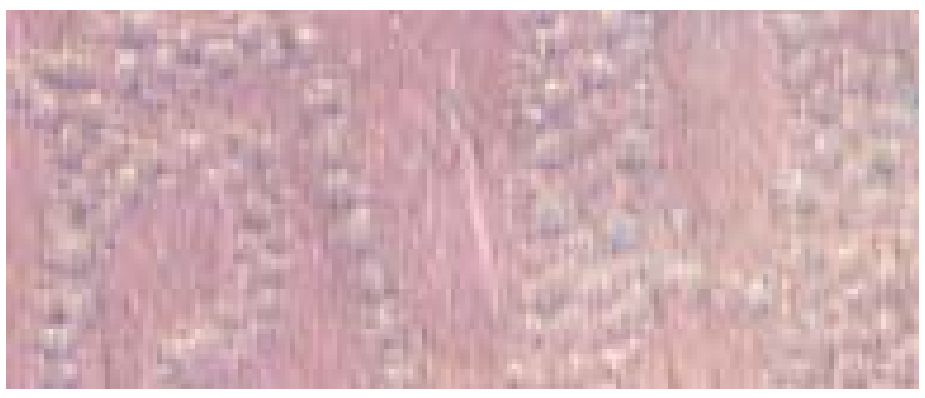

Blister packs seen with this fake sticker are printed with batch number 010401.

Counterfeit Artesunate – Type 6

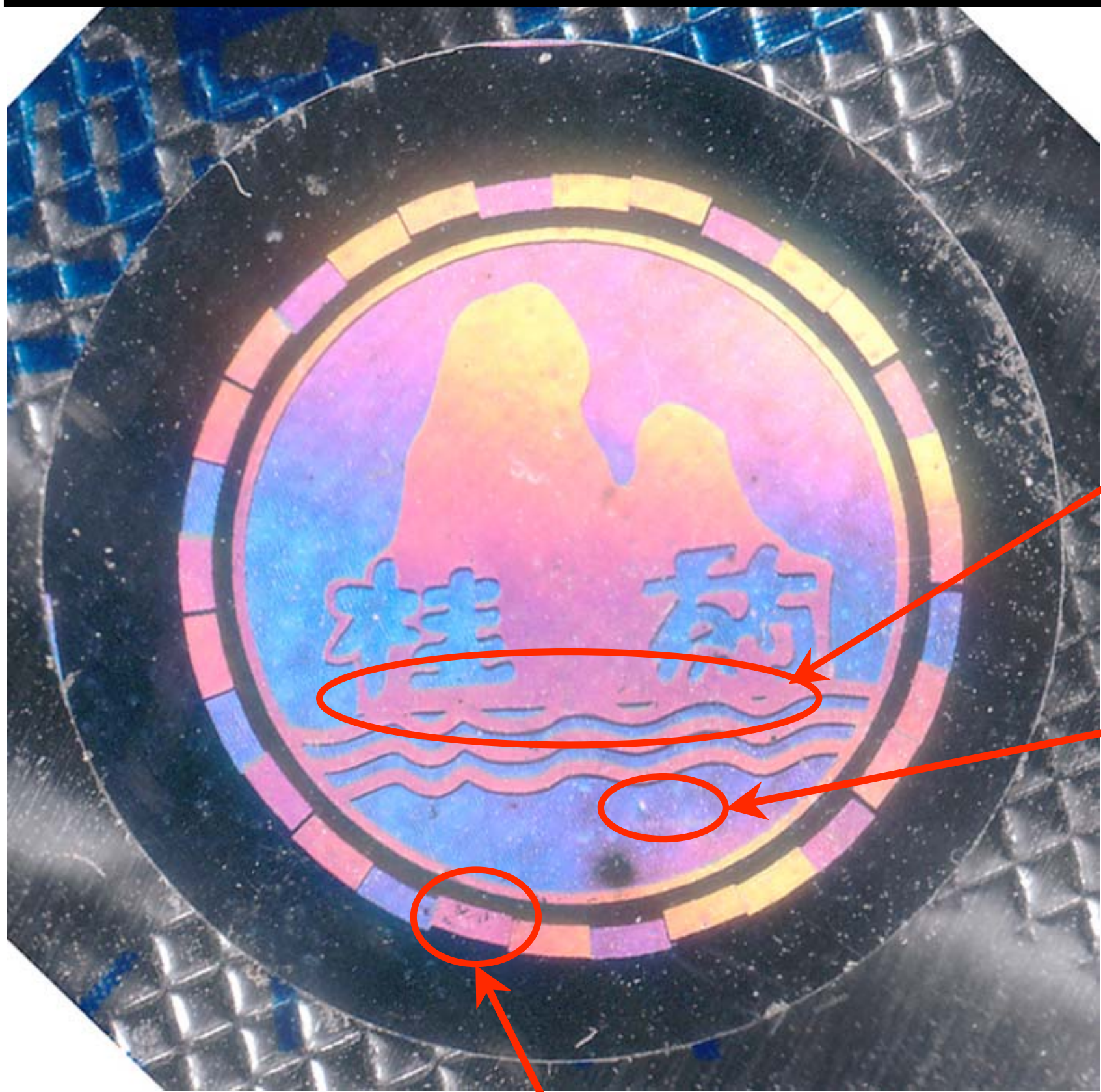

Well crafted hologram - but the junction between the mountains and the waves differs from that depicted on the genuine hologram. There is an indistinct legend to the right of centre, beneath the waves

Crescents, rather than a continuous blank line, between mountain and waves

Indistinct writing below waves, ending in '…MA', to the right of centre

Blister packs seen with this fake hologram are printed with batch numbers 030101 and 010101

Ring 'blocks' out of alignment

Counterfeit Artesunate – Type 7

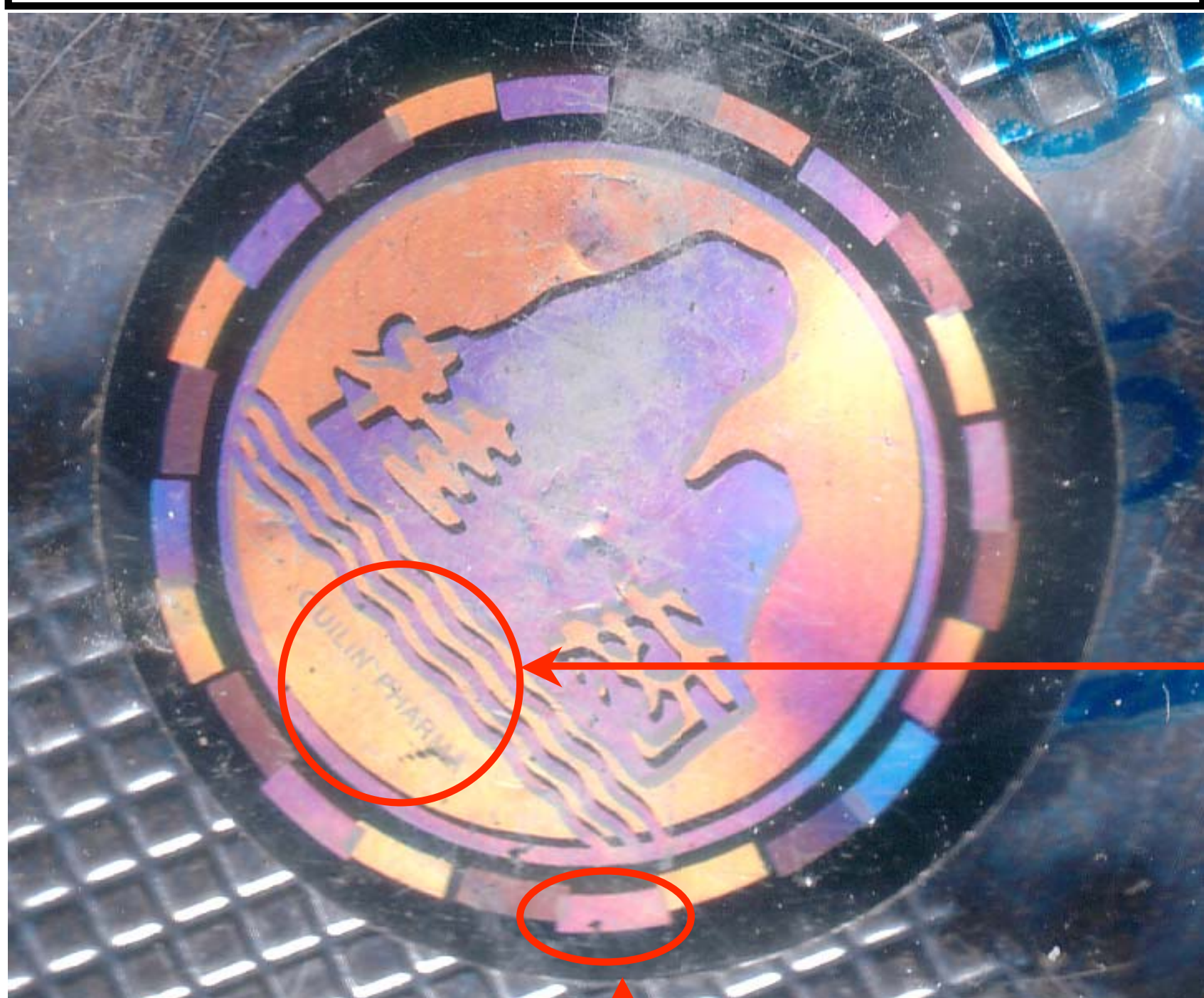

Well crafted hologram - with 'GUILIN PHARMA' written below the waves but in an abnormally large font

'Guilin Pharma' legend present but the letters are of larger font than those on the genuine hologram and can be read with the naked eye. Letters are ~ 0.3 mm high.

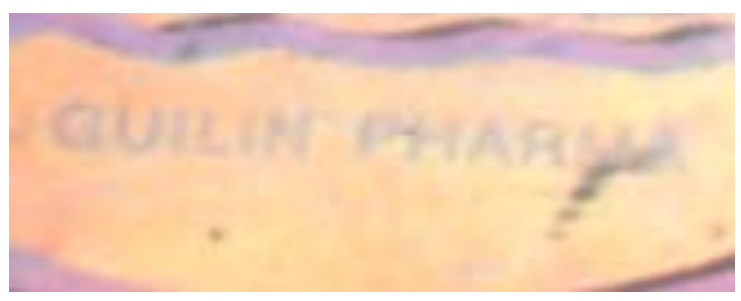

Ring 'blocks' out of alignment

Blister packs seen with this fake hologram are printed with batch number 000801.
Date of expiry is normally 3 years after date of manufacture. This type has an abnormally long lifetime of nine years

Counterfeit Artesunate – Type 8

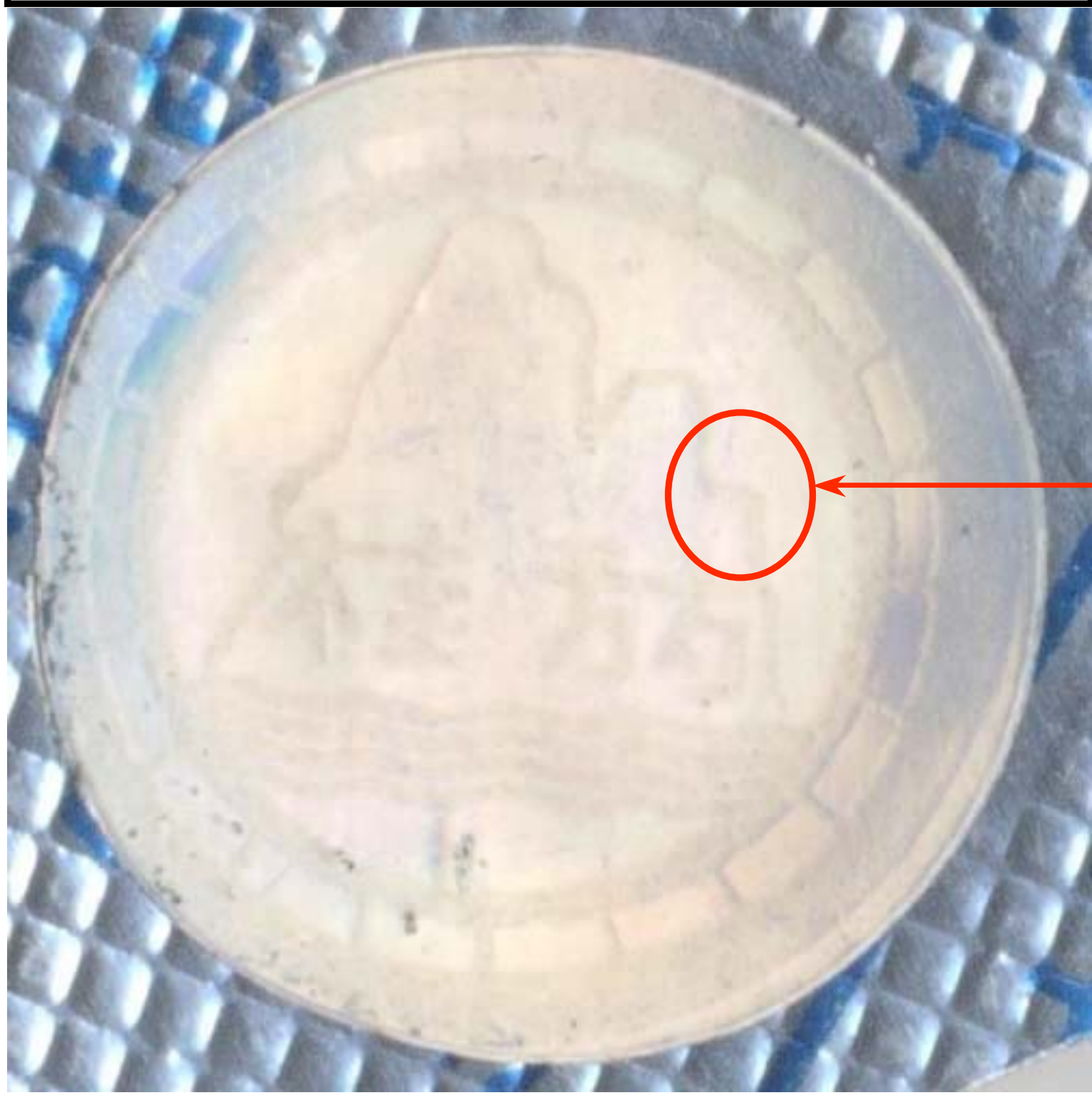

Sticker - similar to 'Type 2' but with 'plateau' on right side of mountain outline and a very acute angle between the two mountain peaks. 'Artesunate Tablet' printed on blisterpack

Plateau

Blister packs seen with this fake sticker are printed with batch numbers 980201, 010401 or 040201
Some have manufacture and expiry abbreviations printed in capitals 'MFG' and 'EXP' in contrast to the genuine product which uses 'Mfg' and 'Exp'.

Counterfeit Artesunate – Type 9

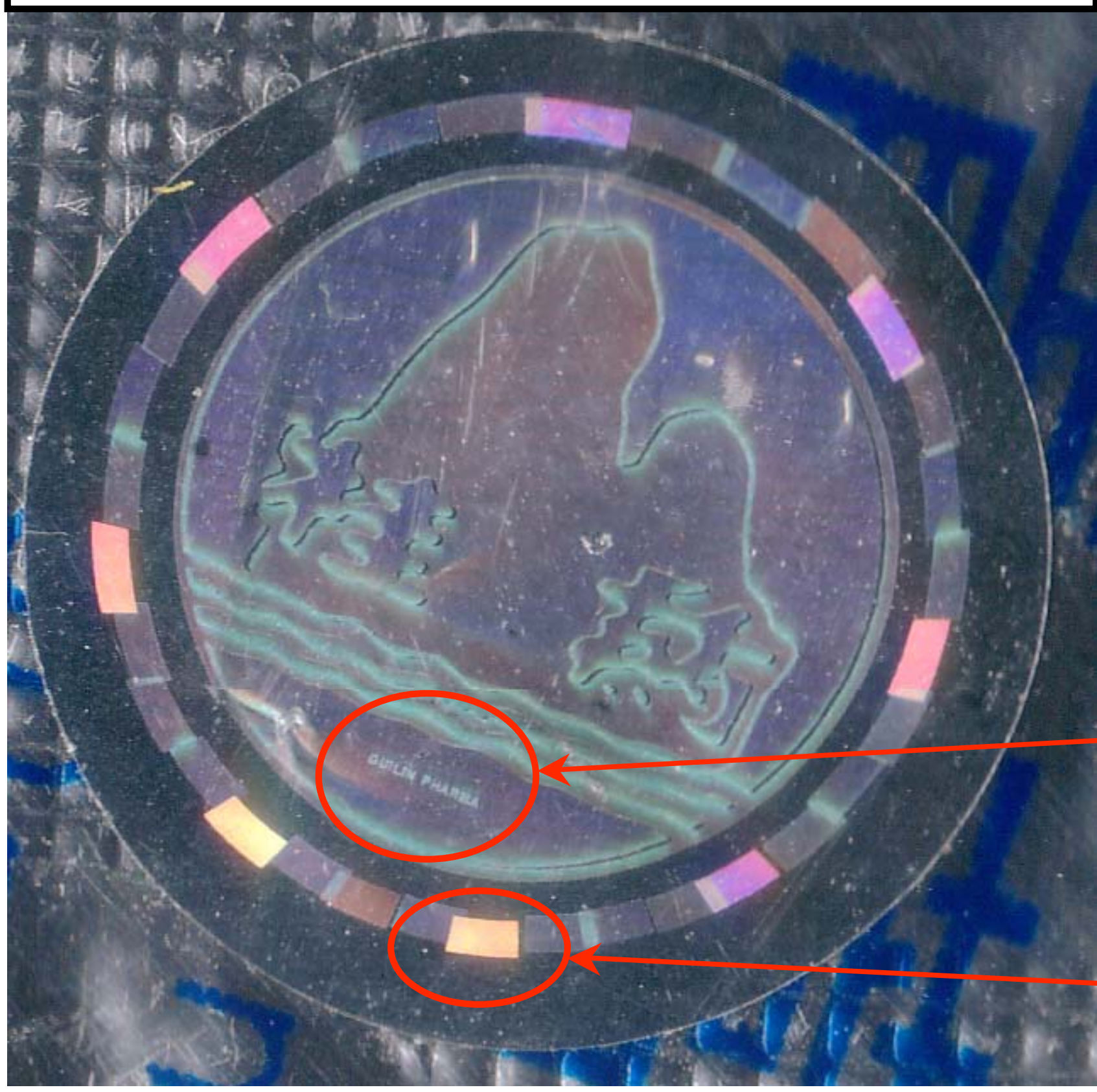

Well crafted hologram, similar to 'Type 7' but Guilin Pharma legend below waves is in a smaller font, similar to the font on the genuine product. 'Artesunate Tablets' printed on the blisterpack.

Traces of artesunate found in tablets

Blister packs seen with this fake hologram are printed with batch number 040101, 4010 or 040502

'Guilin Pharma' legend

Ring 'blocks' out of alignment

Counterfeit Artesunate – Type 10

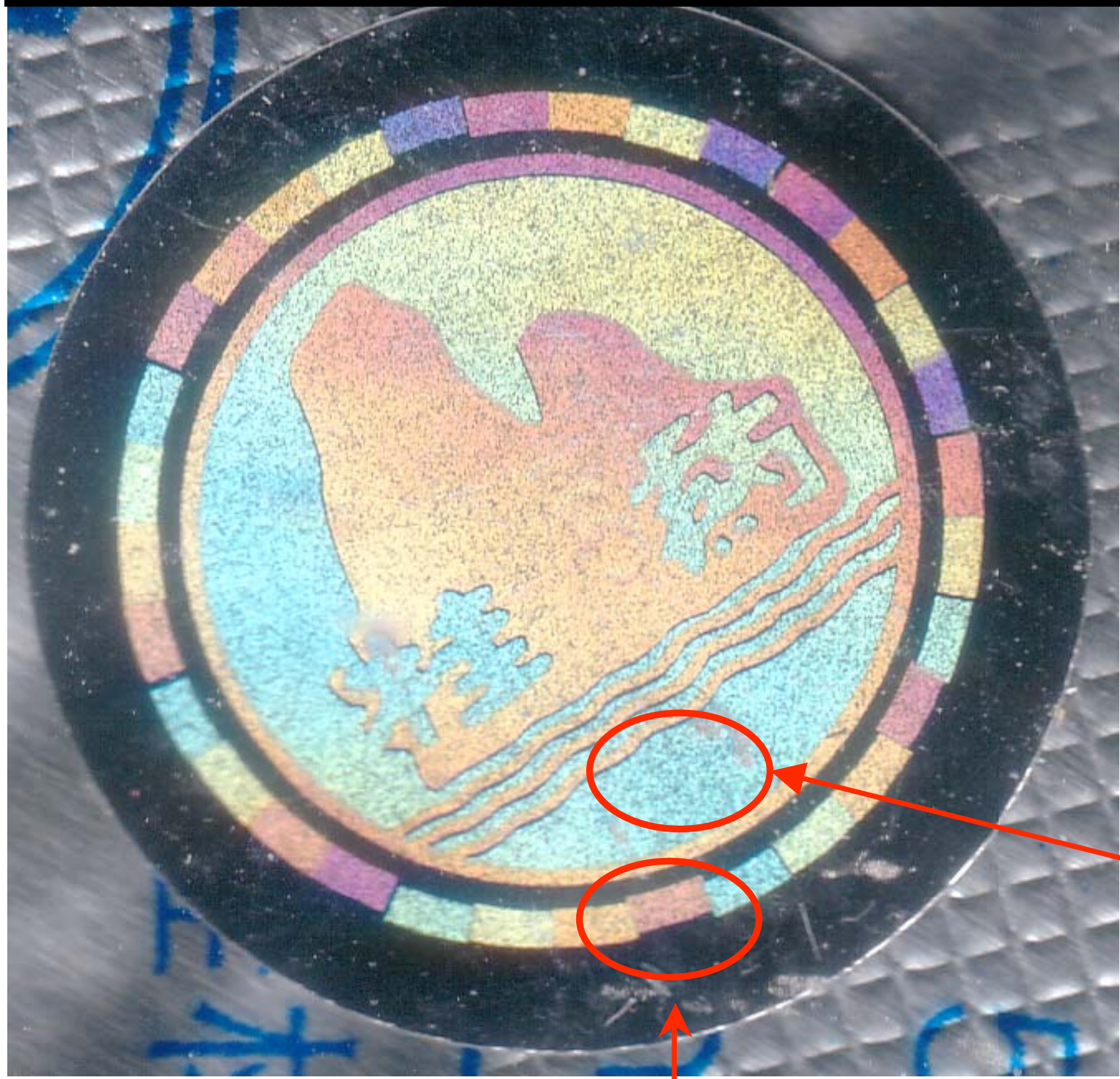

Well crafted hologram, similar to ‘Type 4’, but has ‘X-52’ visible under UV light on hologram (see below). The X-52 is however stamped across the middle, rather than the base, of the mountains. ‘Artesunate Tablets’ printed on blisterpack.

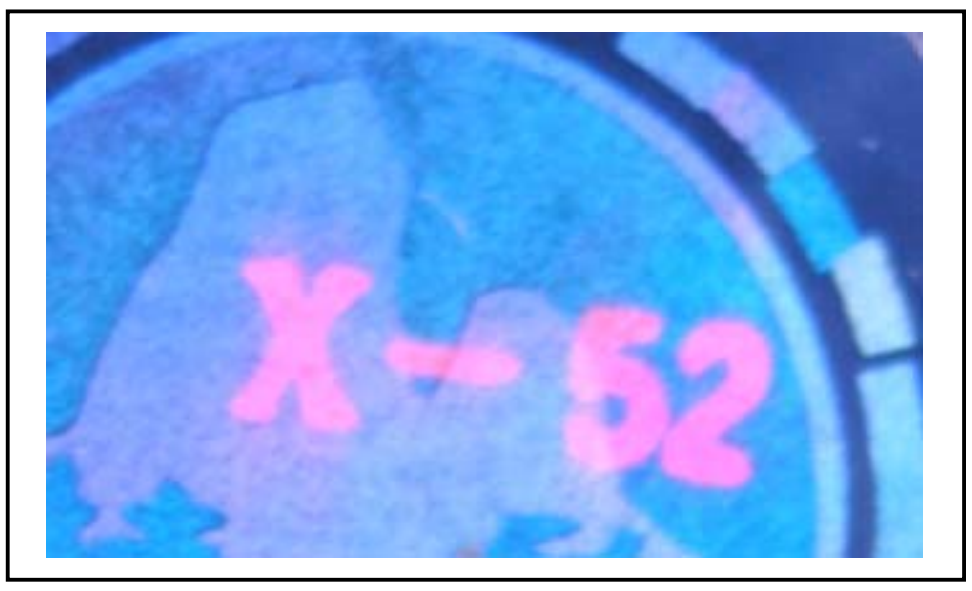

No Guilin Pharma legend

Blister packs seen with this fake hologram are printed with batch number 040301

Ring ‘blocks’ out of alignment

Counterfeit Artesunate – Type 11

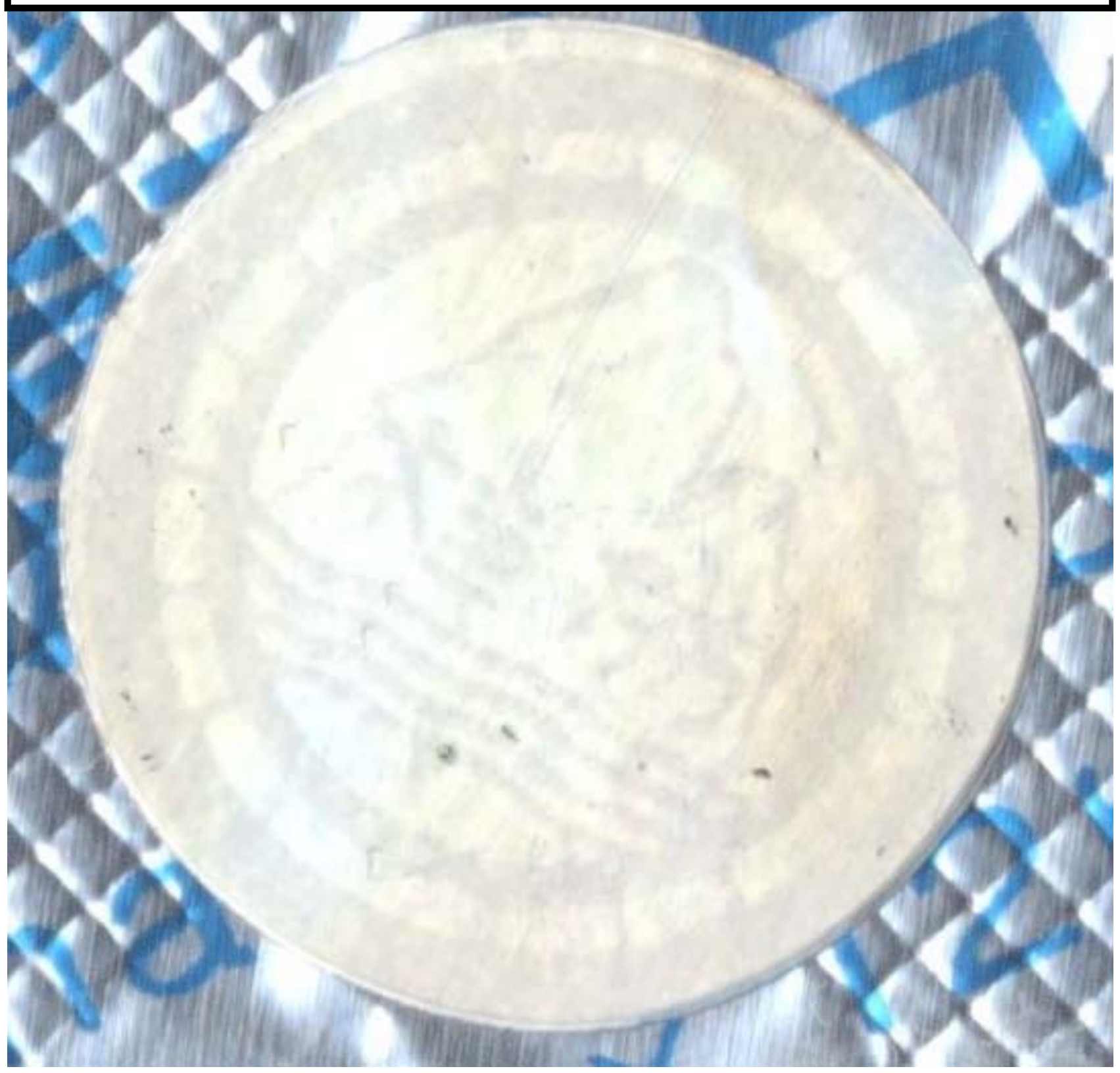

Sticker, similar to ‘Type 2’ and ‘8’ but mountain outline differs and poorly manufactured. ‘Artesunate Tablet’ printed on blisterpack.

Blister packs seen with this fake sticker are printed with batch numbers 010401, 020402, 030301

Manufacture and expiry abbreviations are printed in capitals ‘MFG’ and ‘EXP’ in contrast to the genuine product which uses ‘Mfg’ and ‘Exp’.

Counterfeit Artesunate – Type 12

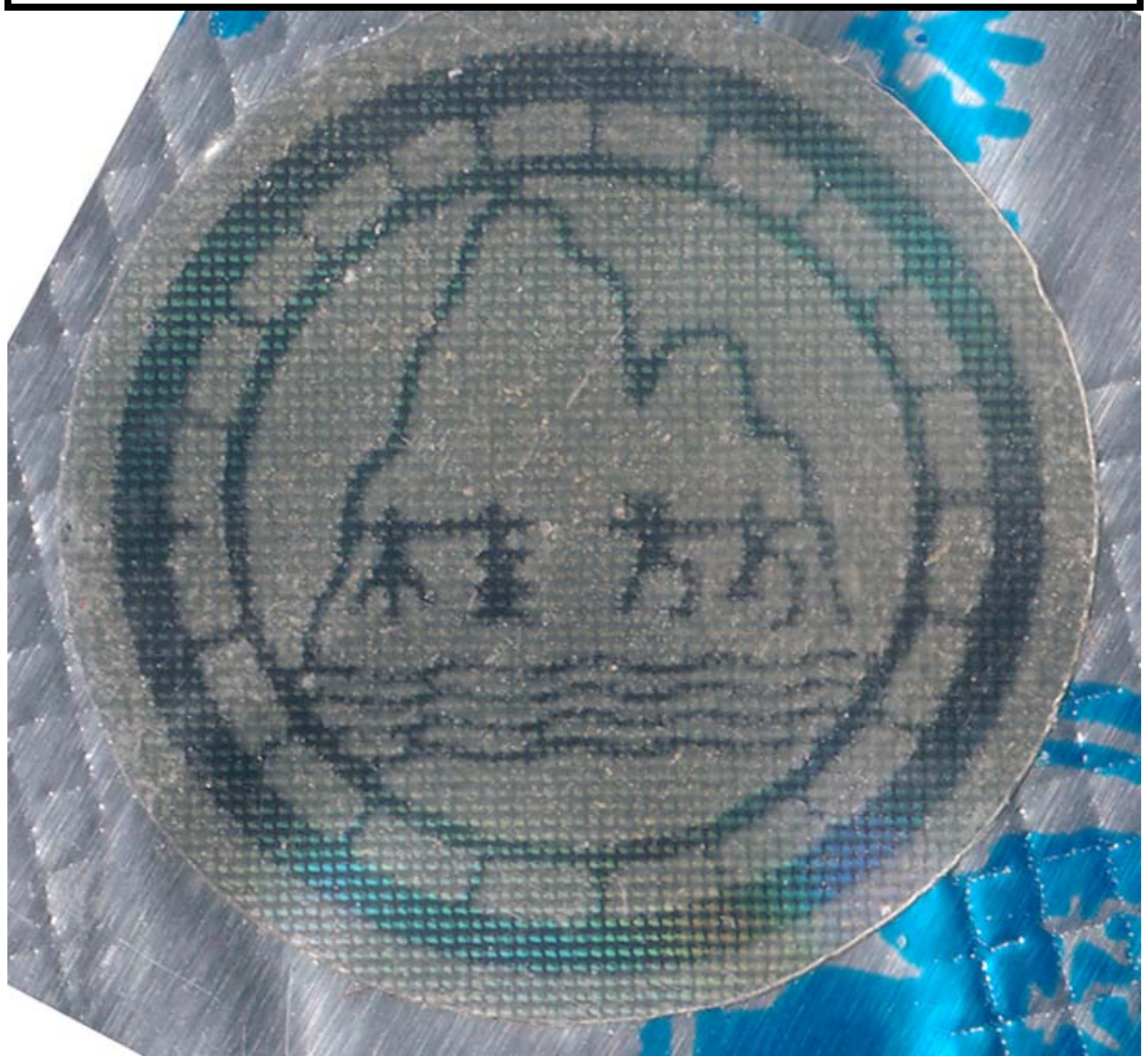

Sticker, with similar mountain outline to 'Type 8' and '11' but with a grid of large dots and a 'cloth' like appearance 'Artesunate Tablets' printed on blisterpack.

Blister packs seen with this fake sticker are printed with batch number 040201.

Manufacture and expiry abbreviations are printed in capitals 'MFG' and 'EXP' in contrast to the genuine product which uses 'Mfg' and 'Exp'.

References concerning the counterfeit artesunate problem

1. Aldhous P. Murder by medicine. *Nature* 2005; **434**: 132–136
2. Archer P. Queen's Plan to Combat Fake Drugs. *The Scotsman* (UK) 2nd Dec 2004
3. Carpenter J. A Review of Drug Quality in Asia with focus on anti-infectives. USP. http://www.uspdqi.org/pubs/other/ANEReview.pdf.
4. Carpenter J. A Matrix of Drug Quality Reports on USAID-assisted countries by the USP Drug Quality and Information Program, 2004. http://www.uspdqi.org/pubs/other/GHC-DrugQualityMatrix.pdf
5. Crampton T. Fake malaria pills haunt Asians. *International Herald Tribune*, 25th August 2003, page 1
6. Dondorp AM, PN Newton, M Mayxay *et al*. Fake antimalarials in Southeast Asia are a major impediment to malaria control: multinational cross-sectional survey on the prevalence of fake antimalarials. *Trop Med Int Hlth* 2004; **9**: 1241–6
7. Green MD, Dwight LM, Wirtz RA, White NJ. A colorimetric field method to assess the authenticity of drugs sold as the antimalarial artesunate. *J Pharm Biomed Anal* 2000; **24**: 65 –70.
8. Green MD, Mount DL, Wirtz RA. Authentication of artemether, artesunate and dihydroartemisinin antimalarial tablets using a simple colorimetric method. *Trop Med Int Health*. 2001; **6:** 980–2
9. Newton PN, Proux S, Green M *et al*. Fake artesunate in southeast Asia. *Lancet* 2001; **357**: 1948–1950.
10. Newton PN, Dondorp A, Green M, Mayxay M, White NJ. Counterfeit artesunate antimalarials in SE Asia. *Lancet* 2003; **362**: 169.
11. Rozendaal J. Fake antimalaria drugs in Cambodia. *Lancet* 2001; **357**: 890
12. Rozendaal J. Fake antimalarials circulating in Cambodia. *Mekong Malaria Forum* 2000; **7**: 62-69.
13. Phanouvong S, Reiss S, Smine A. Why be concerned about the quality of antimalarials and ARV Drugs. Poster at 7th International Congress on AIDS in Asia and the Pacific, Kobe, Japan, July 1-5, 2005. http://www.uspdqi.org/pubs/other/whyDrugQuality.pdf
14. Smine A, Phancouvong S, Chanthap L, Tsuyuoka R, Nivana N, Blum N (2004) Antimalarial Drug Quality in Mekong Countries. Poster at International Conference on the Use of Medicines held March 30–April 2 2004, Chiang Mai, Thailand. http://www.uspdqi.org/pubs/other/AntimalarialPoster.pdf
15. USP. Fake antimalarials found in Yunnan Province, China, 2004. http://www.uspdqi.org/pubs/other/FakeAntimalarialsinChina.pdf

Wellcome Trust Oxford SE Asian Tropical Medicine Research Units: 4th edition

Compiled by Paul Newton 4th April 2006

With very many thanks to the numerous people who assisted with this project, particularly Facundo Fernandez (Georgia Tech) and Mike Green (CDC) for chemical analysis and all who collected samples

Please distribute : Copies available from paul@tropmedres.ac or http://www.jr2.ox.ac.uk/ndm/Tropical_Medicine/News.htm
